# The striatal compartments, striosome and matrix, are embedded in largely distinct resting-state functional networks

**DOI:** 10.3389/fncir.2025.1514937

**Published:** 2025-05-16

**Authors:** Alishba Sadiq, Adrian T. Funk, Jeff L. Waugh

**Affiliations:** Division of Pediatric Neurology, Department of Pediatrics, University of Texas Southwestern Medical Center, Dallas, TX, United States

**Keywords:** compartment, diffusion tractography, functional connectivity, functional MRI, matrix, striatum, striosome, structural connectivity

## Abstract

The striatum is divided into two interdigitated tissue compartments, the striosome and matrix. These compartments exhibit distinct anatomical, neurochemical, and pharmacological characteristics and have separable roles in motor and mood functions. Little is known about the functions of these compartments in humans. While compartment-specific roles in neuropsychiatric diseases have been hypothesized, they have yet to be directly tested. Investigating compartment-specific functions is crucial for understanding the symptoms produced by striatal injury, and to elucidating the roles of each compartment in healthy human skills and behaviors. We mapped the functional networks of striosome-like and matrix-like voxels in humans *in-vivo*. We utilized a diverse cohort of 674 healthy adults, derived from the Human Connectome Project, including all subjects with complete diffusion and functional MRI data and excluding subjects with substance use disorders. We identified striatal voxels with striosome-like and matrix-like structural connectivity using probabilistic diffusion tractography. We then investigated resting-state functional connectivity (rsFC) using these compartment-like voxels as seeds. We found widespread differences in rsFC between striosome-like and matrix-like seeds (*p* < 0.05, family wise error corrected for multiple comparisons), suggesting that striosome and matrix occupy distinct functional networks. Slightly shifting seed voxel locations (<4 mm) eliminated these rsFC differences, underscoring the anatomic precision of these networks. Striosome-seeded networks exhibited ipsilateral dominance; matrix-seeded networks had contralateral dominance. Next, we assessed compartment-specific engagement with the triple-network model (default mode, salience, and frontoparietal networks). Striosome-like voxels dominated rsFC with the default mode network bilaterally. The anterior insula (a primary node in the salience network) had higher rsFC with striosome-like voxels. The inferior and middle frontal cortices (primary nodes, frontoparietal network) had stronger rsFC with matrix-like voxels on the left, and striosome-like voxels on the right. Since striosome-like and matrix-like voxels occupy highly segregated rsFC networks, striosome-selective injury may produce different motor, cognitive, and behavioral symptoms than matrix-selective injury. Moreover, compartment-specific rsFC abnormalities may be identifiable before disease-related structural injuries are evident. Localizing rsFC differences provides an anatomic substrate for understanding how the tissue-level organization of the striatum underpins complex brain networks, and how compartment-specific injury may contribute to the symptoms of specific neuropsychiatric disorders.

## Introduction

The striatum serves as the primary subcortical connection hub, playing an essential role in a diverse array of motor and cognitive functions (Haber et al., 2006; Jung et al., 2014; Graybiel and Grafton, 2015). Abnormalities in both the structure and function of the striatum have been associated with a range of neurological conditions, including Parkinson disease (Albin et al., 1989), Huntington disease (Rosenblatt and Leroi, 2000), autism spectrum disorder (ASD) (Schuetze et al., 2016), and schizophrenia (Chakravarty et al., 2015). The human striatum is composed primarily of medium spiny neurons (MSNs) that develop in two spatially segregated tissue compartments, the striosome and matrix. Compartment-specific functions demonstrated in animals suggest that compartment-selective injury could lead to distinct symptoms in human diseases. Over a dozen human diseases exhibit compartment-selective injury or a pattern of symptoms that suggests a compartment-selective injury (Crittenden and Graybiel, 2011; Marecek et al., 2024). Recent studies extended the understanding of compartment-specific disease mechanisms beyond the Crittenden and Graybiel (2011), suggesting that additional disorders, including ASD (Kuo and Liu, 2020; Waugh et al., 2025), substance use disorders (Salinas et al., 2016), and certain neuropsychiatric conditions such as mood disorders in early Huntington disease (Waldvogel et al., 2012; Hedreen et al., 2024) or anxiety disorder (Karunakaran et al., 2021), also exhibit differential effects on the striosome and matrix compartments, indicating that the number of diseases with compartment-selective pathologies is likely higher than previously proposed. To the best of our knowledge, these hypothesized compartment-symptom associations have not been tested in living humans: tissue must be *ex-vivo* (for fixation and immunohistochemical staining) to distinguish the striatal compartments, limiting the exploration of human diseases with striatal pathologies. Although the striosome and matrix were recognized in brain tissue 4 decades ago, the lack of tools to identify the compartments in living organisms has made it difficult to study striosome- and matrix-specific functions.

The striosome and matrix compartments exhibit distinct pharmacologic characteristics that suggest a basis for separate functional roles. The striosome is enriched with mu-opioid receptors (MORs) and has lower levels of calbindin, a neurochemical profile that aligns them with the modulation of dopamine-related signaling, crucial for emotional regulation and reward processing (McGregor et al., 2019). Limbic-related regions, such as the prelimbic cortex (PL), basolateral amygdala (Ragsdale and Graybiel, 1988; McGregor et al., 2019), and anterior cingulate (Eblen and Graybiel, 1995) selectively project to the striosome. The striosome, but not the matrix, selectively inhibits the dopaminergic projection neurons of the substantia nigra pars compacta (Crittenden et al., 2016; McGregor et al., 2019), emphasizing their involvement in reward-based learning and behavioral reinforcement. Eblen and Graybiel demonstrated in macaque that limbic cortices selectively project to the striosome, and that these projections are organized somatotopically within the striatum (Eblen and Graybiel, 1995). Likewise, the striosome plays a crucial role in integrating inputs from these limbic regions, which are essential for regulating behavior and emotional responses. Prager and Plotkin (2019) identified a differential response to dopamine between striosome and matrix compartments, noting that dopamine release is modulated differently in each compartment, potentially influencing distinct behavioral outcomes, and contributing to the pathophysiology of neuropsychiatric disorders. These findings suggest that the striosome may also be involved in the pathophysiology of neuropsychiatric disorders. These findings underscore that striosome function is closely linked to regulation of nigral dopamine signaling and coordination of activity among limbic regions, emphasizing the role of the striosome in emotional and motivational processing (Crittenden and Graybiel, 2011).

In contrast, the matrix is characterized by higher calbindin and lower MOR expression, is selectively targeted by projections from somatomotor cortices (primary sensory cortex, primary motor cortex, supplementary motor area; Gerfen, 1984; Donoghue and Herkenham, 1986) and projects to the primary output nuclei of the striatum, the globus pallidus interna (Giménez-Amaya and Graybiel, 1990) and substantia nigra pars reticulata (Desban et al., 1989). This structural organization highlights the role of the matrix in motor control and sensorimotor integration, distinct from the striosome. Considering the compartments’ distinct inputs and projections, relatively segregated distributions within the striatum, opposing responses to dopaminergic regulation (Prager and Plotkin, 2019), and selective susceptibility to metabolic injury (Burke and Baimbridge, 1993), there are multiple anatomic bases to propose different functions for striosome and matrix. The differential functions of the striatal compartments are further evidenced by the striosome’s role in stereotypic behaviors (Lewis and Kim, 2009), task engagement (Friedman et al., 2015), and reinforcement learning, particularly in encoding reward prediction errors (Barto, 1995; Houk et al., 1995) and valence discrimination (Friedman et al., 2020). In contrast, the matrix compartment does not significantly influence these functions, suggesting a more specialized role for striosome in these aspects of behavior and learning (Graybiel and Matsushima, 2023).

We previously demonstrated (Waugh et al., 2022) that differential structural connectivity (probabilistic diffusion tractography) distinguishes striatal voxels with striosome-like and matrix-like patterns of structural connectivity. This method replicates the compartment-specific structural connectivity demonstrated through decades of injected tract tracer studies in animals (Funk et al., 2023; Funk et al., 2024), and is highly reliable in living humans, with a 0.14% test-retest error rate (Waugh et al., 2022). These findings support the notion that the human striatum is anatomically organized into distinct compartments, each characterized by unique patterns of structural connectivity. Striatal compartmental organization is not merely structural, however, but also has functional implications (Donoghue and Herkenham, 1986; Flaherty and Graybiel, 1993; Eblen and Graybiel, 1995). Specifically, these studies highlight how corticostriatal connectivity is directed primarily through one striatal compartment, suggesting a potential basis for distinct functional processing of biased corticostriate projections. Although compartment-specific functions are only partially explored in humans, research in animals provides evidence that striosome and matrix have distinct functional roles. For example, the striosome is specifically involved in decision-making under threat and in the formation of negative-valence memories (Saka and Graybiel, 2003; Xiao et al., 2020; Nadel et al., 2021). These findings suggest that the striosome may play a primary role in processing emotionally salient information and guiding behavior in response to negative stimuli. However, despite these insights, a significant gap remains in our understanding of the compartment-specific functions of the striatum, particularly in humans. Moreover, it is unclear how each compartment contributes to more complex or nuanced behaviors, and whether functional distinctions observed in animal models are directly applicable to human behaviors. Further research is needed to delineate the specific roles of striosome and matrix in human cognitive and emotional processing, such as how the compartments interact within larger neural networks.

The human brain achieves complex and contingent regulation of functions in part by modulating interacting and competing networks of connected regions (Menon, 2011). Understanding cognitive and behavioral functions depends on the structure of large-scale brain networks (Bressler and Menon, 2010). Among the many stable intrinsic brain networks, Menon (2011) proposed a “triple-network model” that highlighted the interplay of activation and regulation among three fundamental neurocognitive networks: the default mode network (DMN), salience network (SN), and frontoparietal network (FPN). Further, selective functional abnormalities in the triple-network are characteristic of specific neuropsychiatric disorders (Menon, 2011). Resting-state fMRI studies have consistently identified reduced DMN activity in Alzheimer disease (AD; Binnewijzend et al., 2012; Zhong et al., 2014; Li et al., 2017), major depressive disorder (MDD; Wei et al., 2015), and ASD (Wang et al., 2021). Specific types of disruptions in triple network connectivity are associated with particular neuropsychiatric disorders.

Gaining insight into how the striosome and matrix compartments regulate human brain networks can improve our understanding of the triple-network model and its effects on cognition and behavior. The present study highlights a novel assessment of resting-state functional connectivity (rsFC) in the striosome-like and matrix-like compartments of the striatum in living humans and explores the influence of each compartment on both whole-brain networks and the triple-network. Our examination included a relatively large adult cohort, comprising 674 healthy individuals of both sexes and diverse racial backgrounds. To the best of our knowledge, this is the first study investigating the functional connectivity differences between the striatal compartments in human subjects. Our findings suggest that striosome-like and matrix-like voxels are embedded in largely distinct functional networks. Therefore, compartment-selective injury or maldevelopment may underlie the network derangements and specific symptoms of neurodevelopmental disorders that involve the striatum.

## Materials and methods

**FIGURE 1:**
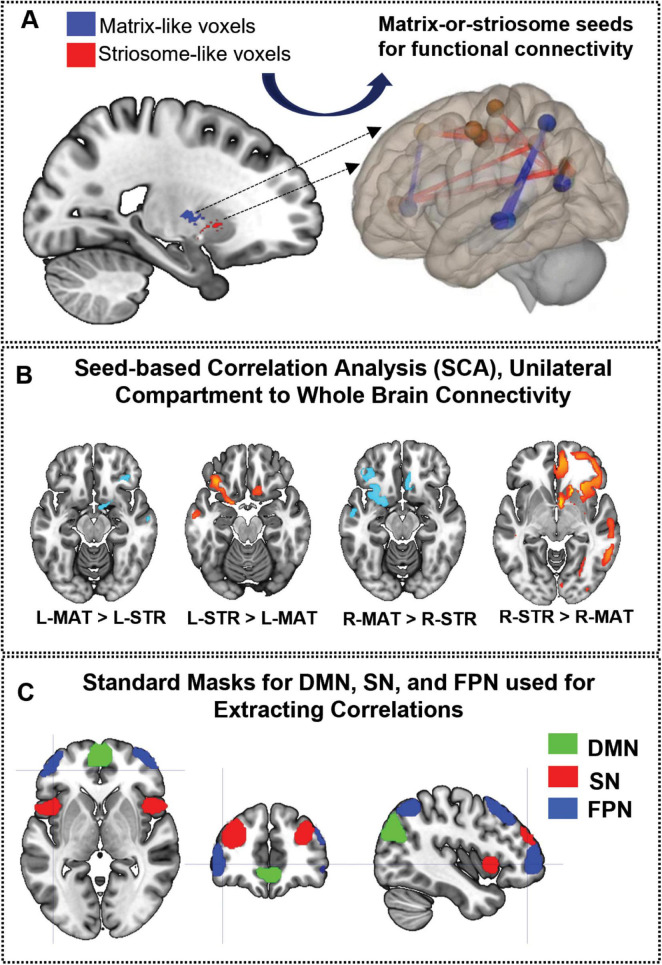
Framework for the investigation of compartment-specific differences in resting-state functional connectivity. **(A)** This sagittal section of the brain illustrates striosome-like (red) and matrixlike (blue) voxels, defined by differential structural connectivity. These voxels were used as seeds for functional connectivity analysis, identifying regions where matrix-like voxels show greater connectivity than striosome-like voxels or vice versa **(B)**, with lighter-hued colors indicating stronger differences. **(C)** Illustrates the nodes utilized to define the triple network: default mode network (DMN—green), salience network (SN—red), and frontoparietal network (FPN—blue). L-MAT, left matrix; LSTR, left striosome; R-MAT, right matrix; R-STR, right striosome.

**FIGURE 2:**
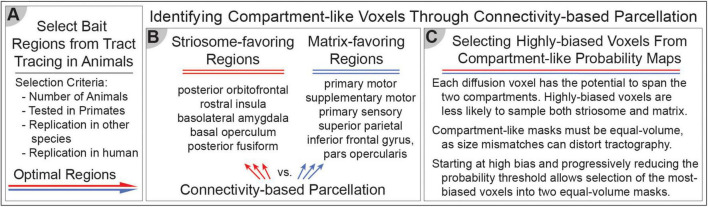
Differential structural connectivity can identify striosome-like and matrix-like striatal voxels. **(A)** The selection of bait regions is based on injected tract-tracing studies in animals, identifying key regions with biased structural connectivity toward one compartment. **(B)** These identified regions serve as targets for connectivity-based parcellation. We identified five striosome-favoring and five matrix-favoring regions with highly biased connectivity to serve as “bait” for connectivity-based parcellation. **(C)** Selecting the most-biased voxels from each probability distribution allows us to generate equal-volume masks that serve as the seeds for functional connectivity in subsequent experiments. These methods enable the investigation of compartment-specific functional networks, integrating structural and functional connectivity approaches.

In Figures 1, 2, we summarize the methodological approaches for investigating compartment specific rsFC in living humans. Figure 1 outlines the foundational approach, while Figure 2 details the generation of compartment-specific striatal masks that serve as seeds for functional connectivity analyses. Briefly, we utilized differential structural connectivity to parcellate the striatum into voxels with striosome-like and matrix-like structural connectivity profiles, generating individualized striatal masks for each subject and hemisphere. These masks follow the spatial distribution, relative abundance, and extra-striate structural connectivity patterns identified through animal and human histology (Waugh et al., 2022; Funk et al., 2023; Funk et al., 2024). The Linux code and anatomical masks necessary for generating striatal parcellations is available here: github.com/jeff-waugh/Striatal-Connectivity-based-Parcellation.

### Study population

The data utilized in this study were sourced from the Human Connectome Project S1200 Release (HCP; Van Essen et al., 2013), which contained behavioral and MRI data from 1,206 healthy young adults. The HCP excluded subjects with significant past neurologic, psychiatric, or neurodevelopmental histories (with the exception of attention deficit disorder that resolved in childhood). Likewise, the study excluded subjects whose siblings were diagnosed with significant neurodevelopmental disorders (e.g., autism spectrum disorder, ASD) or neuropsychiatric diseases, which may have reduced the incidence of subclinical neuropsychiatric symptoms. We selected subjects that had full complements of diffusion MRI and resting-state fMRI data, then eliminated subjects with a history of substance or alcohol use disorders (defined by DSM-5 criteria). We excluded subjects if they had any history of cocaine, hallucinogen, cannabis, nicotine, opiate, sedative, or stimulant drug use in their lifetime. We excluded subjects if they met the DSM criteria for Alcohol Abuse or Alcohol Dependence, or if they averaged > 4 drinks per week over the year preceding their scan. This exclusion criteria produced a cohort of 674 healthy adults (mean age: 28.8 years, *SD* = 3.7 years). Three hundred and ninety-one participants were female; 283 were male. All participants gave written informed consent (Van Essen et al., 2012).

### Acquisition of MRI data

All individuals were scanned at 3T using MRI sequences that were harmonized across multiple sites. The resting-state fMRI data were collected across four runs, each lasting approximately 15 min. Two runs occurred within one session, and the other two occurred within a separate session. Within each session, oblique axial acquisitions alternated between phase encoding in a right-to-left (RL) direction for one run and in a left-to-right (LR) direction for the other run. Participants’ eyes were open and fixated on a crosshair. In this study, we employed the resting-state session 1 for both LR and RL acquisitions, concatenating 1,200 timepoints for each session. Consequently, the resulting concatenated image comprises 2,400 timepoints. Other acquisition parameters included: echo time (TE) = 33 ms, repetition time (TR) = 720 ms, flip angle = 52°; 72 slices; 2.0 mm isotropic voxels; acquisition time = 14 min and 33 s. Diffusion tensor imaging (DTI) data for S1200 subjects was acquired at 1.25 mm isotropic resolution using 200 directions (14 B_0_ volumes, 186 volumes at non-colinear directions) with the following parameters: repetition time = 3.23 s; echo time = 0.0892.

### Striatal parcellation

We previously described a technique for parcellating the human striatum into compartments with striosome-like and matrix-like patterns of connectivity *in-vivo* (Waugh et al., 2022). This is a striatum-centered example of connectivity-based parcellation, which has been utilized for decades to identify brain structures based on differential structural connectivity (Behrens et al., 2003). We refer to parcellated striatal voxels as “striosome-like” or “matrix-like” due to the inferential nature of this method, and to remind readers that these voxels are not the equivalent of striosome and matrix tissue identified through immunohistochemistry. Striatal parcellation uses structural connectivity findings from decades of injected tract tracer investigations in mice, rats, cats, dogs, and non-human primates (summarized in Waugh et al., 2022) to identify regions with compartment-selective projections. Note that none of these studies mapped connectivity with the nucleus accumbens, to the best of our knowledge. Therefore, we performed striatal parcellation only on the neostriatum, not the nucleus accumbens. From these biased regions, identified through animal histology and our prior MRI-based mapping in humans, we selected five striosome-favoring regions and five matrix-favoring regions, as competing targets (“bait”) for quantitative probabilistic tractography (the first step in connectivity-based parcellation). These bait regions matched those we have utilized in prior striatal parcellations in different cohorts (Funk et al., 2023): posterior orbitofrontal cortex, rostral insula, basolateral amygdala, basal operculum, and the posterior fusiform cortex (striosome-favoring); supplementary motor cortex, primary motor cortex, primary sensory cortex, superior parietal cortex, and caudodorsal inferior frontal gyrus, pars opercularis (matrix-favoring).

We performed all tractography in native diffusion space with the FSL utility *probtrackx2*, utilizing standard parameters: curvature threshold = 0.2; steplength = 0.5 mm; number of steps per sample = 2,000; number of samples per seed voxel = 5,000; distance correction, to prevent target proximity from influencing connection strength. For connectivity-based parcellation we engaged classification targets mode, which produced paired striatal maps (one for streamlines that reached striosome-favoring bait regions, one for streamlines that reached matrix-favoring bait regions). At each striatal voxel we then compared the number of streamlines that reached striosome-favoring bait regions *vs.* matrix-favoring bait regions. The ratio of these seed-to-target counts provided an assessment of the compartment-like bias of each striatal voxel (the final step in connectivity-based parcellation). The relative connectivity to these two groups of bait regions defined the compartment-like bias for each striatal voxel, creating a map of striosome-like and matrix-like connection probability that was unique to each individual and hemisphere.

Since each diffusion voxel has the potential to include both striosome and matrix, many striatal voxels have only modest compartment-like connectivity bias. We selected the most-biased voxels with the goal of identifying voxels that were minimally “diluted” by the other compartment. For each individual and hemisphere, we started at the most-biased end of the probability distribution (complete bias toward one compartment) and iteratively reduced the bias threshold until the mask matched our target volume. The target volume for these masks was equal to 13% of the starting striatum mask (equivalent to the volume 1.5 standard deviations above the mean in a Gaussian distribution). Mismatches in the volume of target masks can skew probabilistic tractography, so we ensured that striosome-like and matrix-like masks were equal for each subject and hemisphere. These high-bias voxels from each distribution represented striosome and matrix for subsequent experiments. We previously demonstrated that this 1.5 SD volume threshold is sufficient to recapitulate the relative abundance, spatial distribution, and selective connectivity of striosome and matrix that were demonstrated in histology (Waugh et al., 2022; Funk et al., 2023). Notably, our MRI-based method yields connectivity biases that match the structural networks identified in non-human primates: in primary and supplementary motor cortex (Parthasarathy et al., 1992; Inase et al., 1996) in macaque; primary sensory cortex in squirrel monkey (Flaherty and Graybiel, 1993); posterior orbitofrontal cortex in macaque (Eblen and Graybiel, 1995; Haber et al., 1995); and globus pallidus interna and externa in squirrel monkey (Giménez-Amaya and Graybiel, 1990, Flaherty and Graybiel, 1993). In the current study we used these parcellated striatal voxels as seeds for rsFC with either whole-brain networks (excluding the striatum), or with nodes in the triple-network.

Striosome branches are embedded in the surrounding matrix (Graybiel and Ragsdale, 1978; Holt et al., 1997); in any given plane, the striosome appears as separated “islands” while the matrix is a contiguous “sea.” We used the *fsl-cluster* command to assess the relative segregation of voxels within striosome-like and matrix-like distributions, using a bias threshold of *P* > 0.87 to select for the most representative voxels. We restricted our volume comparison to the first/largest cluster, as it included roughly 95% of non-segregated voxels. Similarly, striosome- and matrix-like voxels are uniquely located in each individual; while the striosome is enriched in rostral, medial, and ventral parts of the striatum (Graybiel and Ragsdale, 1978; Goldman-Rakic, 1982; Donoghue and Herkenham, 1986; Ragsdale and Graybiel, 1990; Desban et al., 1993; Eblen and Graybiel, 1995; Waugh et al., 2022), at any particular location one may find striosome-like or matrix-like voxels. We measured the location of each striosome-like and matrix-like voxel to assess whether our parcellated voxels matched the compartment-specific location biases established in histology. For each subject and hemisphere, we identified the cartesian position (x, y, and z planes) of each compartment-like voxel. We then measured the within-plane and root-mean-square distances from that voxel to the centroid of the nucleus (caudate or putamen) in which the voxel resided. Finally, we measured the volume of compartment-like voxels (*P* > 0.55) within 2 mm coronal planes (rostral to caudal), separately in caudate and putamen. This allowed us to compare the relative abundance of each compartment-like distribution throughout the striatum. Intersubject variation in the size and position of the striatum led planes at the rostral and caudal margins to miss the striatum completely for some subjects. We excluded planes that had volume data for < 50% of our cohort (one rostral plane, two caudal planes for each nucleus).

We previously demonstrated that slight shifts in voxel location were sufficient to eliminate any compartment-like biases in structural connectivity, indicating that compartment-specific connectivity biases were specific for those precise locations, not for the “neighborhood” in which they reside (Funk et al., 2023). We hypothesized that compartment-specific biases in functional connectivity would also be dependent on precise voxel location. We jittered the location of our compartment-like voxels by ± 0–3 voxels, at random and independently in each plane. For the first 100 subjects in this cohort, we tested the cartesian position of each striosome- and matrix-like voxel before and after jitter to assure that while individual voxels shifted position, the mean location of these randomized masks had the same topographic organization as our compartment-specific masks. No voxels in the randomly shifted masks were reselected from either original compartment-like mask. We assessed the striosome-like and matrix-like bias (*P* > 0.55) within our original highly biased masks (which we used as the seeds for rsFC) and these location-shifted voxels (which we utilized as a negative control for rsFC).

We performed a second round of probabilistic tractography (streamline mode) as a *post-hoc* assessment of the spatial segregation of streamlines seeded by our high-bias compartment-like voxels. This round was seeded by (A) our high-bias, matched-volume striosome-like and matrix-like voxels, or (B) by the location-shifted voxels described above. Streamlines were restricted to the ipsilateral hemisphere but were otherwise unbounded in their connectivity. We utilized the same *probtrackx2* parameters that we utilized for striatal parcellation, described above. For each native-space streamline bundle, we imposed an amplitude threshold to isolate the core of the bundle (the uppermost 25% of voxels; Waugh et al., 2019). We then assessed the overlap of compartment-like bundles within each individual using the Dice similarity coefficient (DSC). We compared the mean DSC for our precisely selected compartment-like voxels and the location-shifted voxels.

### Analysis of resting-state functional connectivity

Functional connectivity measures were calculated between striatal seed masks (striosome-like, matrix-like, or randomized location) and all other non-striatal voxels in the brain. For each subject, we extracted the residual BOLD signal time course from the seed mask by averaging the signal across all voxels within that mask. Next, we computed the Pearson correlation coefficient between this mean seed time course and the time course of every voxel in the rest of the brain, generating compartment-specific voxel-wise correlation maps. Correlation values were transformed to fisher’s *z*-score to normalize the distribution of data. The rsFC between each compartment-like seed and each component of the triple-network was evaluated using a series of network-specific masks from the connectivity (CONN) toolbox (Whitfield-Gabrieli and Nieto-Castanon, 2012). We evaluated the three limbs of the triple-network: the default mode network (DMN), comprising the medial prefrontal cortex (mPFC), bilateral lateral parietal cortex, and posterior cingulate cortex (PCC, which also included parts of the precuneus); the salience network (SN), consisting of the dorsal anterior cingulate cortex (dACC), bilateral anterior insula (AI), bilateral rostral prefrontal cortex (PFC), and bilateral supramarginal gyrus (SMG); and the frontoparietal network (FPN), including the bilateral dorsolateral prefrontal cortex (dlPFC) and bilateral posterior parietal cortex (PPC). All 15 regions of interest (ROIs) were sourced from the CONN toolbox. The predefined masks in the CONN toolbox (to identify the DMN, SN, and FPN) were generated using an independent component analysis (ICA) on resting-state fMRI data from 497 participants of the Human Connectome Project (Van Essen et al., 2012). These ICA-derived masks have been validated in previous studies for their reproducibility and accuracy in capturing the intrinsic connectivity patterns of these networks (Boots et al., 2022; Muehlhan et al., 2024). The CONN toolbox provides a user-friendly platform for functional connectivity analysis and includes these standardized network templates as a resource. By using these validated masks, our study leverages well-established network definitions, ensuring that our connectivity analyses are consistent with prior literature and appropriate for investigating the DMN, SN, and FPN networks. By using publicly available ROIs, we minimized the possibility of bias that could result from manually selecting ICAs derived from our own data set, strengthening our external validation. Each ROI’s connectivity with striosome-like and matrix-like voxels was assessed independently, rather than performing a full-factorial analysis.

### N-1 (leave one out) analyses

We previously demonstrated that this set of bait regions, which included the anterior insula, produced robust striatal parcellations (Waugh et al., 2022). However, it is not possible to use a region to parcellate the striatal compartments and then accurately measure connectivity between the compartments and that same region. To avoid this issue when assessing connectivity with the SN (which included the anterior insula as a node), we performed a *post-hoc* validation in which we excluded the anterior insula from the set of bait regions and parcellated the striatum again using the remaining four striosome-favoring regions and original five matrix-favoring regions. We used this “leave one out” parcellation to seed rsFC only for measures that included the anterior insula, referred to here as an “N-1 analysis” (Funk et al., 2023).

We performed nine additional N-1 striatal parcellations, one for each bait region. For each N-1 parcellation we generated equal-volume high-bias masks (described above, Methods 2.3). We averaged each parcellation across our 674 subjects. We then subtracted each N-1 average volume from the original “5 *vs.* 5” average volume to reveal the location and amplitude of the left-out region’s contributions to compartment-like bias (Avg_5_ *_*vs*_.*_5_–Avg_5_ *_*vs.*_*_4(A)_; Avg_5_ *_*vs*_.*_5_–Avg_5_ *_*vs.*_*_4(B)_, etc.) We performed binary segmentation at each striatal voxel using the FSL tool *find_the_biggest*, which identified large-scale striatal zones where a particular bait region was the strongest influence on compartment-like connectivity. Within each of these large zones, we assessed the volume at the 50th percentile of the maximum amplitude. We adjusted the amplitude thresholds of each N-1 distribution to generate refined somatotopic zones with 50–100 voxels per hemisphere. For each zone, we matched volume between left and right hemispheres within a few voxels. We did not utilize cluster-forming algorithms to define these somatotopic zones. We measured the volume of biased voxels (*P* > 0.55) within the striosome-like and matrix-like distributions (5 *vs.* 5 parcellation).

### Statistical analysis

We assessed the intra-striate position, volume, clustering, and compartment-like bias of our parcellated striatal voxels using a series of two-tailed paired-samples or unequal-variance *t*-tests. We compared the volume within streamline bundles seeded by compartment-like voxels using two-tailed paired-samples *t*-tests. We assessed compartment-favoring bias within 10 somatotopic zones (one for each bait region) using ANOVA. We considered compartment, region, hemisphere, and subject as explanatory factors, and included the interaction of compartment and region as a nuisance parameter. We assessed the volume of striosome-like voxels within somatotopic zones using a single-factor ANOVA. We corrected for multiple comparisons within each family of tests (Benjamini and Hochberg, 1995).

Next, we carried out whole-brain rsFC mapping. Statistical analyses were conducted on the correlation maps generated from striosome- and matrix-like seeds. Each subject contributed four maps, corresponding to connectivity dominated by four distinct seed regions: (1) left-matrix (L-MAT), (2) left-striosome (L-STR), (3) right-matrix (R-MAT), and (4) right-striosome (R-STR). Differences in correlation maps with the rest of the brain (excluding the striatum) were observed.

Finally, we carried out rsFC mapping for the nodes of the DMN, SN, and FPN to assess compartment-specific alterations within the triple-network. These analyses were executed using SPM12 software.^1^ Paired *t*-tests were employed to compare the compartment-specific correlation maps (connectivity with striosome-like seeds *vs.* connectivity with matrix-like seeds) for each network-specific ROI. We modeled age and sex as covariates because both factors have been shown to influence brain connectivity patterns. Including these covariates helps to account for individual variability that might otherwise confound the differences in connectivity between striosome-like and matrix-like voxels. We set a cluster-level threshold of *p* < 0.05, corrected for the family wise error (FWE) rate, indicating a significant difference in rsFC between connectivity seeded by striosome-like and matrix-like voxels. Specifically, after computing voxelwise statistics, an initial uncorrected voxel-level threshold (e.g., *p* < 0.001) was applied to identify clusters of contiguous voxels. Then, using random field theory, we determined the probability of observing each cluster by chance, and only clusters with an FWE-corrected *p*-value of less than 0.05 were considered significant. This method controlled the probability of making false positive errors across the entire brain, increasing the likelihood that our findings were robust against the multiple comparisons inherent in whole-brain analyses.

## Results

### Compartment-like voxels match the anatomic features of striosome and matrix

Prior studies determined that the striosome and matrix comprise approximately 15 and 85% of striatal volume, respectively, in both animal and human histology (Johnston et al., 1990; Desban et al., 1993; Holt et al., 1997; Mikula et al., 2009). We assessed the total number of highly biased (*P* > 0.87) striosome-like and matrix-like voxels for each subject. Striosome-like voxels made up 5.9% of highly biased voxels, while matrix-like voxels made up 94.1%. Note that at this high threshold, most striatal voxels have middling-bias (a blend of striosome- and matrix-like connectivity) and thus are not counted in this metric. At the lowest threshold for defining compartment-like bias (*P* > 0.55), striosome-like voxels made up 22.7%, matrix-like voxels made up 67.9%, and indeterminate (mixed-bias) voxels made up 9.4% of striatal volume. Note that our striatal mask did not include the caudate tail, which is comprised almost entirely of matrix (Bernácer et al., 2008; Mikula et al., 2009), which may have reduced the relative abundance of matrix-like voxels as a fraction of whole-striatal volume. These results align with human and animal histology, showing far more matrix-like than striosome-like voxels.

The relative intra-striate location of striosome and matrix is highly consistent across species (mouse, rat, cat, macaque, human), with striosome enriched in the rostral, ventral, and medial striatum and the matrix enriched in the caudal, dorsal, and lateral striatum (Graybiel and Ragsdale, 1978; Goldman-Rakic, 1982; Donoghue and Herkenham, 1986; Ragsdale and Graybiel, 1990; Desban et al., 1993; Eblen and Graybiel, 1995; Waugh et al., 2022). Our voxel-by-voxel location analysis recapitulated this compartment-specific location bias in both hemispheres. In the caudate we found that matrix-like voxels were more lateral (0.88 mm, *p* = 1.3 × 10^–78^), caudal (7.9 mm, *p* < 1 × 10^–260^), and dorsal (8.2 mm, *p* < 1 × 10^–260^) than striosome-like voxels. In the putamen we found that matrix-like voxels were shifted more lateral (2.4 mm, *p* < 1 × 10^–260^), caudal (5.6 mm, *p* < 1 × 10^–260^), and dorsal (6.3 mm, *p* < 1 × 10^–260^) than striosome-like voxels. Striosome-like voxels in the caudate were 10.8 mm medio-rostro-ventral to the centroid (RMS distance), while matrix-like voxels were 11.5 mm latero-caudo-dorsal to the centroid. Striosome-like voxels in the putamen were 9.8 mm medio-rostro-ventral to the centroid, while matrix-like voxels were 7.5 mm latero-caudo-dorsal to the centroid.

**FIGURE 3:**
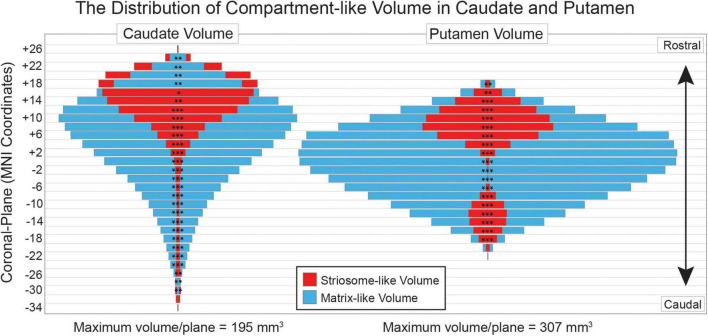
Compartment-like voxels match the location and relative abundance of striosome and matrix in human tissue. Striosome-like volume (red) is concentrated in the rostral caudate (left) and putamen (right), while matrix-like volume (blue) is distributed throughout the rostral-caudal extent of both nuclei. For each plane, the smaller-volume bar is placed in front of the larger-volume bar—red (striosome-like) in front of blue (matrix-like) for all putaminal planes and most caudate planes. Coronal planes are numbered according to MNI convention. Significance assessed with *t*-tests, unequal variance: *p < 0.05; **p < 10^– 5^; ***p < 10^– 50^. Significance threshold corrected for family wise error.

In tissue, striosome and matrix differ in relative abundance and typical location. The relative abundance of each compartment also varies by location. We assessed the volume of the striosome-like and matrix-like distributions (*P* > 0.55) in coronal planes spanning the rostral-caudal extent of the striatum (Figure 3). Of the 48 coronal planes with sufficient data for comparison, all had significant differences in compartment-like volume (*p*-values ranged from 0.046 to < 10^–305^, FWE-corrected for multiple comparisons). Matrix-like volume was larger than striosome-like volume for all 19 putaminal planes (matrix-like volume as a percent of all compartment-like volume, range: 63–99%). Matrix-like volume was larger than striosome-like volume for 22 of 28 caudate planes (range: 52–96%). In tissue, striosome volume is predominantly found in the head of the caudate (Mikula et al., 2009). Similarly, we found that the only planes with substantially more striosome- than matrix-like volume were in the head of the caudate: in the four rostral-most planes, striosome-like volume exceeded matrix-like volume by 5.5–12.6% (range, *p*-values, 3.8 × 10^–8^–1.4 × 10^–29^). The caudal-most planes of the caudate had very low volume for both matrix-like and striosome-like voxels (mean, < 5 voxels for each compartment). Therefore, while the volume of striosome-like voxels was larger in two caudal planes (−30 and −28), these differences made a minimal contribution to total compartment-like volume.

In histologic sections, each striosome branch is surrounded by contiguous matrix tissue (Graybiel and Ragsdale, 1978; Holt et al., 1997). In contrast, our diffusion MRI voxels sampled the striatum in a rigid grid that did not align with each individual’s uniquely positioned striosome, leading to partial volume effects, mixing of compartments within most voxels, and blurring of striosome-like signal across neighboring voxels. At this resolution it was impossible to resolve the connectivity biases of single striosome branches. Despite this limitation, striosome-like voxels were 18-fold less likely than matrix-like voxels to occur in clusters. The mean volume of the largest cluster in the striosome-like distribution had 14.9 voxels [SEM + 0.87; 95% CI (13.2, 16.6)]. In contrast, the mean volume of the largest matrix-like cluster was 262 voxels [SEM + 5.7; 95% CI (250, 273)]; paired samples *t*-test, striosome-like *vs.* matrix-like volume, *p* = 7.6 × 10^–250^). As in tissue, striosome-like voxels are largely segregated from one other, while matrix-like voxels are likely to occupy large, contiguous clusters.

**FIGURE 4:**
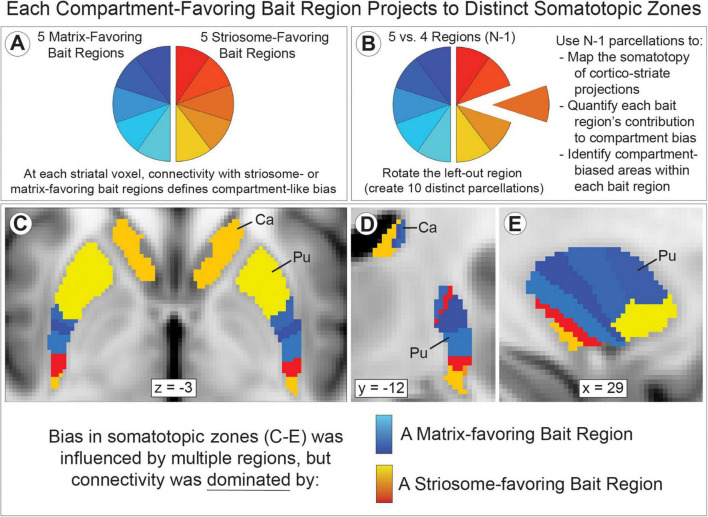
Striatal parcellation utilizing all 10 bait regions **(A)** maps compartment-like bias throughout the striatum. N-1 parcellation [**(B)**; only 9 regions, leaving one out] also identifies compartment-like bias but provides a way to map and quantify the contributions of each bait region. Subtracting these parcellations (All-10 minus N-1) reveals the influence of the left-out region. We performed binary striatal parcellation, comparing each of our 10 N-1 segmentations to identify the largest contributor to bias at each voxel. In axial **(C)**, coronal [**(D)**, left hemisphere], and sagittal planes [**(E)**, left hemisphere], the influence of each bait region followed complex, three-dimensional patterns in both caudate (Ca) and putamen (Pu). Though we parcellated the left and right hemispheres independently, somatotopic zones were highly similar in size, location, and sequence between the hemispheres **(C)**. Zones influenced by matrix-favoring bait regions are shown in shades of blue, while zones influenced by striosome-favoring bait regions are shown in shades of red-yellow. Note that each region influences connectivity outside its somatotopic zone, but less strongly than within its zone. Data is projected on the MNI152_T1_1 mm standard template. Coordinates follow MNI convention.

**FIGURE 5:**
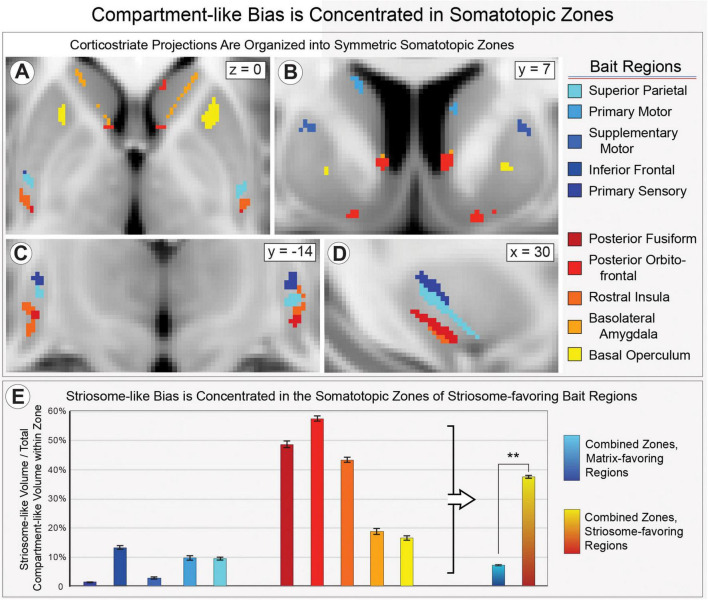
Compartment-specific projections target discrete somatotopic zones in the striatum, in complex 3D patterns that are highly similar between left and right hemispheres, seen in axial **(A)**, coronal **(B, C)**, and sagittal **(D)** views. All masks had similar volume (50–100 voxels); left and right zones for each bait region were matched within a few voxels. Compartment-like volume within these zones **(E)** demonstrated that each somatotopic zone matched the connectivity pattern (near-exclusive matrix or striosome enriched) found in animal histology. Error bars represent the standard error of the mean. **p = 6.5 × 10^– 52^, ANOVA. Data is projected on the MNI152_T1_1 mm standard template. Coordinates follow MNI convention.

Compartment-specific cortico-striate projections are organized somatotopically (Goldman-Rakic, 1982, Ragsdale and Graybiel, 1990, Flaherty and Graybiel, 1993, Eblen and Graybiel, 1995). We mapped the locations where each bait region made the strongest contributions to compartment-like bias, in large somatotopic zones that filled the striatum (Figure 4), and in refined zones that included only the most-biased voxels within each somatotopic zone (Figure 5). Similar to the somatotopic zones mapped through injected tracers in animals (Flaherty and Graybiel, 1993), in humans, cortico-striate projections target specific portions of the striatum in patterns that are highly symmetrical between the hemispheres (Figures 4C–E, 5A–D). Notably, we performed striatal parcellation and defined these somatotopic zones independently in the left and right hemispheres. The high degree of similarity between the hemispheres in these independent measures, and the low spatial variance between individual subjects, suggests that connectivity-based striatal parcellation is driven by specific anatomic differences between striosome-like and matrix-like voxels.

Two regions, the basal operculum and basolateral amygdala, were remarkable for having much broader contributions to compartment-like bias than other regions. The full width at half maximum (FWHM) volume at the 50th percentile for these two regions was 65% larger than the FWHM measures for the other eight regions combined. Basal operculum and basolateral amygdala influenced compartment-like bias to a lesser degree than other regions but were more likely than other regions to influence bias within other somatotopic zones. We described this dispersed pattern of connectivity previously in a separate human MRI cohort (Waugh et al., 2022).

We aimed to learn whether the somatotopic zones identified through our MRI-based method matched the compartment biases identified in animals through injected tract tracers (summarized in Waugh et al., 2022). We measured striosome-like and matrix-like volume within each of these refined somatotopic zones (Figures 5A–D), expressed as the percent of all supra-threshold voxels that were striosome-like (Figure 5E). For somatotopic zones dominated by matrix-favoring regions, striosome-like voxels made up only 7.3% (SEM + 0.24%). For zones dominated by striosome-favoring regions, striosome-like voxels made up 40.0% (SEM + 0.46%). The least-biased striosome-favoring zone, basal operculum, was significantly more likely to include striosome-like volume than the inferior frontal gyrus, the most-biased matrix-favoring zone (16.6% *vs.* 13.4%, respectively; *p* = 4.1 × 10^–4^). We assessed the influence of compartment bias (whether a region was identified as striosome-favoring or matrix-favoring in animals), hemisphere, and subject on striosome-like bias within these somatotopic zones. There was a significant effect of compartment bias on striosome-like volume (F_1_,_13478_ = 232, *p* = 6.5 × 10^–52^). The *R*^2^ value for this model was 0.43. For 10 of 10 bait regions, the compartment bias predicted by animal tract tracing studies matched the bias identified through our MRI-based method.

Streamlines seeded by striosome-like or matrix-like voxels had markedly different volumes of distribution, as assessed by the Dice similarity coefficient (DSC). These streamline bundles had similar total volume (striosome: 8,039 mm^3^, SEM + 28.9; matrix: 8,218 mm^3^, SEM + 28.6) but were largely segregated [DSC: 5.2%; 95% CI (4.9, 5.6)]. The “projectomes” of striosome-like and matrix-like voxels were almost entirely distinct. In contrast, streamlines seeded by striatal voxels in the same “neighborhood” (randomly shifted by + 0–3 voxels in each plane) were 5.5-fold more likely to overlap [DSC: 28.8%; 95% CI (28.0, 29.5)]; paired-samples *t*-test, precise *vs.* location shifted, *p* < 10^–275^). This pattern of segregated volumes was dependent on the precise location of our compartment-like voxels, not the striatal “neighborhood” in which they were located. Striosome-like and matrix-like voxels also differed markedly in the probability of completing a streamline, despite the fact that their seed volumes were exactly matched and they were executed with identical tractographic parameters. The total number of streamlines within the core of the striosome-like bundle was 3.2 × 10^9^ [SEM + 2.9 × 10^7^, 95% CI (3.1 × 10^9^, 3.3 × 10^9^)], which was 48.6% larger than the streamlines seeded by matrix-like bundles [2.2 × 10^9^; SEM + 1.9 × 10^7^; 95% CI (2.1 × 10^9^, 2.2 × 10^9^)]; paired-samples *t*-test, striosome-like *vs.* matrix-like bundles, *p* = 4.7 × 10^–243^. Location-shifted seeds had the opposite pattern: streamlines seeded by voxels adjacent to striosome-like masks had 8.1% *smaller* streamline bundles relative to those seeded by voxels adjacent to matrix-like masks (2.1 × 10^9^ *vs.* 2.3 × 10^9^, respectively). Shifting the location of striosome-like voxels by a few millimeters converted their probability of structural connectivity to one that nearly matched the bundles seeded by matrix-like voxels.

### Hemispheric differences in striosome-like and matrix-like functional connectivity

**FIGURE 6:**
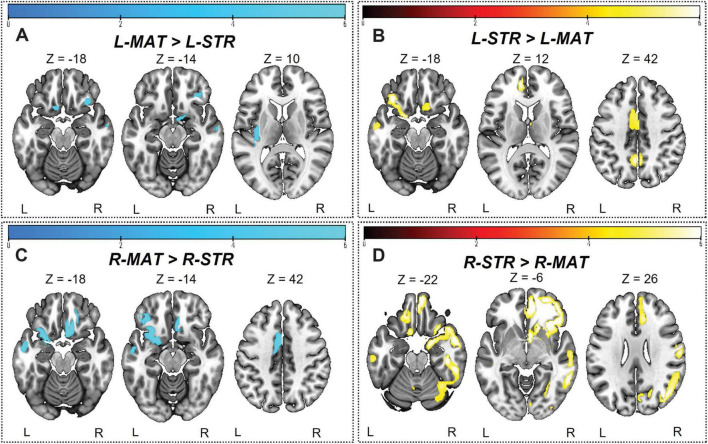
Significant differences between striosome- and matrix-seeded networks in the **(A)** left-matrix (L-MAT), **(B)** left-striosome (L-STR), **(C)** right-matrix (R-MAT), and **(D)** right-striosome (R-STR). Warm colors (red-yellow) indicate regions where striosome-like seeds showed significantly greater rsFC compared to matrix-like seeds; cool colors (blue-light blue) indicate regions where matrix-like seeds showed greater rsFC than striosome-like seeds. Higher *t*-values indicate higher connectivity. Images are displayed in anatomical convention, where the left hemisphere is shown on the left side of the image.

**FIGURE 7:**
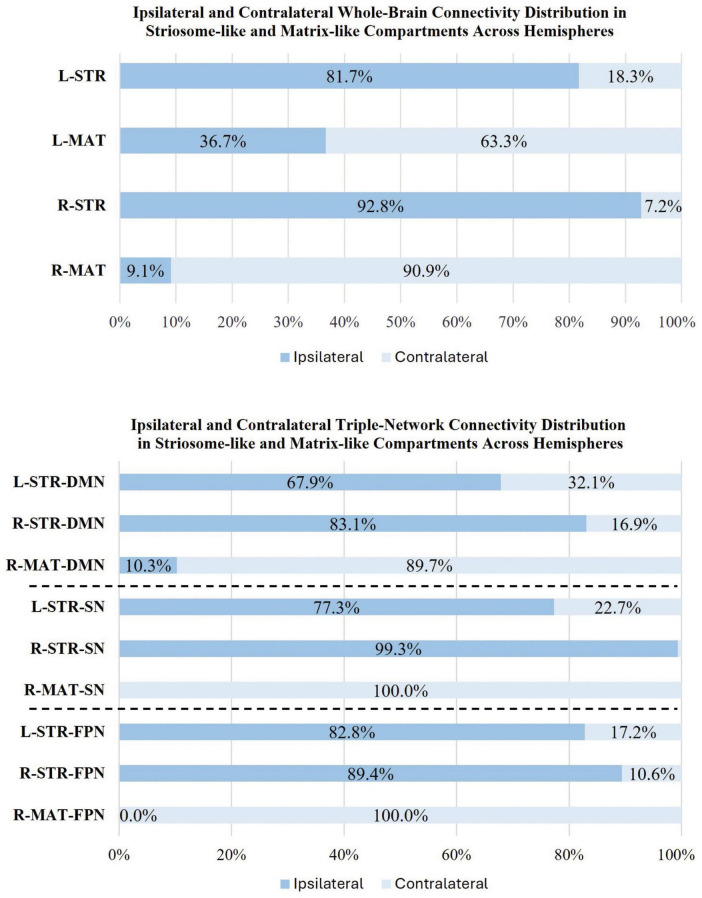
Comparison of ipsilateral and contralateral connectivity patterns in striosome and matrix compartments across hemispheres. The figure shows the distribution of whole-brain **(top panel)** and triple-network (**bottom panel**: DMN, default mode network; SN, salience network; FPN, frontoparietal network) connectivity for ipsilateral and contralateral connections within striosome- and matrix-like compartments. Note that the L-MAT had no significant contrasts for DMN, SN, or FPN, so these comparisons are not shown in this figure.

We observed significant differences in whole-brain rsFC between striosome-like and matrix-like seeds, including notable compartment-specific differences in ipsilateral *vs.* contralateral connectivity (Figures 6, 7). The R-STR exhibited the highest hemispheric bias, with 92.8% of its significant voxels connected within the ipsilateral hemisphere. Conversely, connectivity with the R-MAT was dominated by contralateral connectivity, with 90.9% of its significant voxels in the left hemisphere.

The L-STR also demonstrated a high degree of ipsilateral connectivity, at 81.7%, though less biased than the R-STR. The L-MAT shows a more balanced distribution, with 63.3% of its significant voxels in the ipsilateral and 36.7% in the contralateral hemisphere, indicating moderate interhemispheric communication. Overall, the R-STR and R-MAT regions showed starkly contrasting hemispheric connectivity patterns, while the left hemisphere compartment-like seeds showed the same ipsilateral:contralateral pattern, but with smaller hemispheric bias.

In addition to compartment-specific hemispheric differences in rsFC, we identified rsFC differences in distinct regions in both hemispheres. L-MAT maps showed higher functional connectivity in brain regions involved in sensory-emotional integration and auditory processing, such as the left superior orbitofrontal cortex (sup-OFC), posterior insula (PIns), Heschl’s gyrus (HES), and the right inferior orbitofrontal cortex (Inf-OFC). In contrast, L-STR showed connectivity in relatively few right hemisphere regions, but these were associated with the DMN [precuneus (PCUN), sup-OFC, posterior cingulate cortex (PCC)]. The L-STR also showed higher rsFC to several left hemisphere regions that contribute to cognitive-emotional integration, social cognition, and executive control [middle temporal gyrus (MTG), PCUN, middle and inferior OFC (mid-OFC, sup-OFC), anterior insula (AIns), and middle and anterior cingulate cortex (MCC, ACC)].

Compartment-specific differences between right hemisphere seeds were similar to those in the left hemisphere but were more robust. R-MAT maps showed higher functional connectivity in regions in the left hemisphere: temporal gyrus (TG), middle and superior temporal pole (MTP, STP), OFC, amygdala (AMYG), fusiform (FUS), AIns, postcentral gyrus, MCC, ACC, and PCUN. R-MAT also showed greater connectivity in regions in the right hemisphere: PIns and sup-OFC. In contrast, R-STR demonstrated substantially stronger connectivity, compared to R-MAT, in several right hemisphere regions, including the TG, MTP, and STP, inferior and middle frontal gyri (IFG, MFG) and occipital gyrus regions, including the middle and inferior occipital gyrus (MOG, IOG), cuneus (CUN), AIns, MCC, ACC, angular gyrus (ANG), gyrus rectus, hippocampus (HIPP), para-HIPP, AMYG, lingual (LING), FUS, and several cerebellar subregions (lobules VI, VII, VIII, IX; crus I, crus II). In the left hemisphere, R-STR connectivity was significantly elevated in the superior and middle frontal gyrus (SFG, MFG), ACC, postcentral gyrus, and the region encompassing both the gyrus rectus and the medial orbitofrontal cortex (mOFC), a key area involved in olfactory processing.

R-MAT and R-STR are connected to some of the same regions in opposite hemispheres, reflecting a lateralized, potentially opposing, pattern of functional connectivity. The left TG, MTP, STP, AMYG, FUS, and AIns had significant connectivity with R-MAT, while these same regions in the right hemisphere had significant connectivity with R-STR. This similarity in connectivity suggests that, despite their distinct compartmental roles, R-MAT and R-STR influence parallel networks on opposite sides of the brain, highlighting a unique cross-hemispheric mirroring of their connectivity profiles.

### Influence of precise voxel location on striatal compartmentalization

We have proposed that selecting striatal voxels based on biases in their structural connectivity can replicate the anatomic features of striosome and matrix that were demonstrated through histology. However, an alternate explanation for these biases in connectivity is that cortico-striatum projections are somatotopically organized, independent of compartment-level organization, and our striosome-like and matrix-like voxels are simply reflecting the connectivity biases of their local environment. We set out to determine if our precisely selected voxels, or the neighborhood in which they were embedded, was the primary driver of differences in rsFC. We jittered the location of our striosome- like and matrix-like seed voxels by ± 0–3 voxels, at random and independently in each plane. The average root-mean-square distance shifted by individual voxels was modest: 2.9 voxels for randomized-striosome, 3.1 voxels for randomized-matrix. This measure incorporated the absolute value of shifted distance. The average distance shifted in each plane (which included both positive and negative shifts) was minimal: 0.37 voxels (range across all planes: 0.07–1.2 voxels). As planned, shifting voxels at random led to small but appreciable changes in location for individual voxels, but no meaningful change in the averaged location of whole striatal masks; on average, jittered masks occupied the same “neighborhood” as our precise striosome-like and matrix-like masks.

Since the striatum has 5- to 6-fold more matrix than striosome (based on a tissue ratio of 85:15, respectively), randomly shifting the location of a matrix-like voxel was likely to result in the selection of another matrix-like voxel—albeit a less-biased one, since by definition, our starting masks were the most-biased voxels from each compartment-like distribution. In contrast, shifting the location of a striosome-like voxel was much less likely to select another striosome-like voxel, given their lower abundance. The striosome is concentrated in the rostral-ventral-medial striatum, but even in those areas the likelihood of selecting another striosome-like voxel would be less than half (Figure 3; Holt et al., 1997; Mikula et al., 2009).

Therefore, we hypothesized that shifting the location of matrix-like voxels would modestly decrease matrix-like bias, but shifting striosome-like voxels would markedly reduce striosome-like bias. Indeed, we found that shifting matrix-like voxels decreased their bias from 0.98 to 0.89 [−9.2%; 95% CI (0.089, 0.094)], while shifting striosome-like voxels decreased bias from 0.79 to 0.44 [−34.8%; 95% CI (0.34, 0.35)]. Randomizing the location of striosome-like voxels (shifting their location by only a few millimeters) eliminated all striosome-like bias in structural connectivity, a 3.8-fold larger reduction than when shifting matrix-like voxels (*F*_1_,_1346_ = 116.1, *p* = 5.3 × 10^–26^). This pattern of highly concentrated striosome-like bias is consistent with the architecture of the striosome described in human tissue.

These location-shifted seed masks had no differences in rsFC; when comparing functional correlations between striosome-shifted and matrix-shifted seeds, there were no significant voxels. This is consistent with our prior findings in structural connectivity in a separate diffusion MRI dataset (Funk et al., 2023): small shifts in voxel location are sufficient to erase the compartment-specific biases in connectivity. These results suggest that the rsFC biases we observed are highly dependent on the precise localization of voxels within the striatum, rather than the broader neighborhood in which they reside.

### Default mode network

**FIGURE 8:**
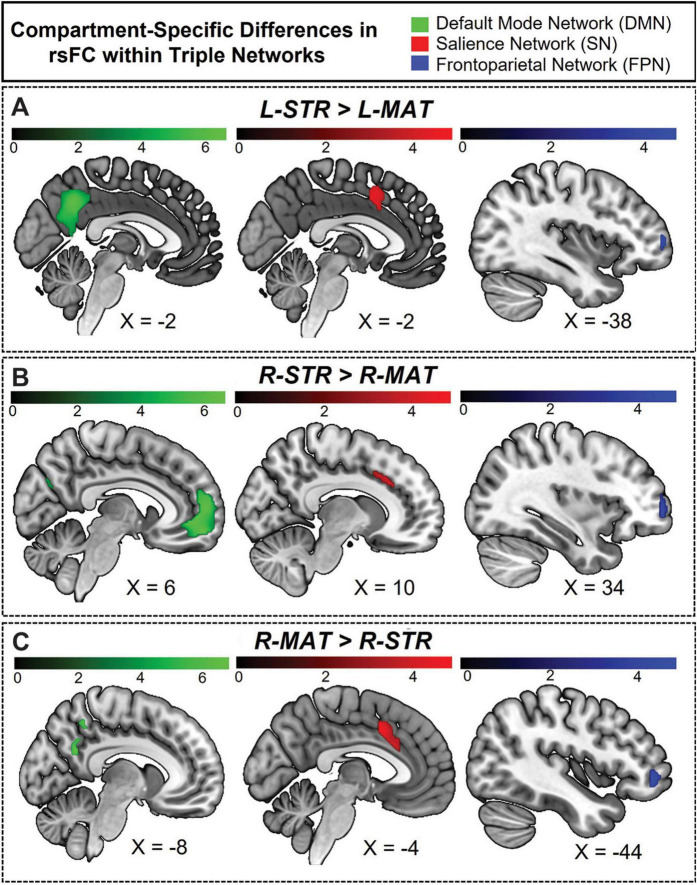
Hemisphere-specific distribution of striosome- and matrix-like compartment rsFC in the triple network. **(A)** Illustrates the regions where the L-STR exhibits stronger functional connectivity than the L-MAT compartment. **(B)** Illustrates the regions where the R-STR has stronger connectivity than the R-MAT. **(C)** Illustrates the regions where the R-MAT has stronger connectivity than the R-STR. Note that the L-MAT > L-STR contrast yielded no significant differences, and therefore is not pictured here. Green indicates regions within the DMN, red indicates regions in the SN, and blue indicates regions in the FPN. The color bars correspond to t-values, with lighter colors reflecting higher *t*-values and stronger connectivity.

We observed substantial, widespread compartment-specific differences in rsFC with the DMN (Figure 8). For L-STR seeds, most significant clusters (67.9%) were within the left hemisphere. Similarly, for R-STR seeds, 83.1% of significant clusters were within the right hemisphere. In contrast, the R-MAT seeds had 89.7% of their significant clusters in the contralateral hemisphere. There were no significant differences for connectivity between DMN and L-MAT. These DMN-specific interactions follow the same fundamental pattern demonstrated for whole-brain networks: striosome-like rsFC is predominantly ipsilateral, while matrix-like rsFC is less robust and predominantly contralateral.

The volume of significant clusters underscores the dominance of striosome-like seeds in influencing the DMN. Significant correlations between L-STR and DMN had a volume of 9,656 mm^3^, while the L-MAT to DMN correlations had a volume of zero. Correlations between R-STR and DMN were substantially larger (14,464 mm^3^) than R-MAT (1,008 mm^3^). Comparing the total number of significant voxels (both hemispheres) between the compartments, striosome-like seeds were 24-fold more likely to correlate with the DMN than matrix-like seeds.

The L-STR dominated connectivity in the precuneus of both hemispheres; L-MAT had no significant correlations. R-STR connectivity was evident in left prefrontal and right middle temporal regions, while R-MAT connectivity was observed in only a small area of the left medial precuneus [MNI coordinates: (−9.7, −53.3, 31.6)]. Significant differences for L-STR-seeded maps were also noted in the ACC, CUN, PCC, and mPFC in the left hemisphere. R-STR-seeded connectivity was significantly higher in the mPFC, MTG, angular gyrus (ANG), CUN, PCUN, and ACC.

Regarding the overlap between hemispheric connectivity, the Dice Similarity Coefficient (DSC) between rsFC seeded by L-STR and R-STR [(L-STR > L-MAT) ∪ (R-STR > R-MAT)], was zero. Overlap between L-STR and R-MAT was minimal (DSC = 0.13). Intriguingly, the sole overlapping region between L-STR and R-MAT was the left precuneus, suggesting that this area may be a unique convergence point that links the otherwise distinct connectivity pattern of striosome-like and matrixlike seeds with the DMN.

### Salience network

The L-STR and R-STR seeds demonstrated predominantly ipsilateral rsFC with SN nodes. Specifically, the L-STR seed exhibited 77.3% connectivity within the left hemisphere, while the RSTR seed showed 99.3% connectivity within the right hemisphere. Regions with significant L-STR–SN connectivity included the left MCC and ACC. The L-MAT showed no significant differences. RSTR–SN connectivity was significant in the right AIns, ACC, and SMG. R-MAT–SN connectivity was significant in the left ACC and AIns, continuing the pattern of contralateral connectivity for matrix. Indeed, 100% of the R-MAT–SN significant clusters were in the left hemisphere.

The volume of significant differences suggests that striosome-like seeds were dominant in influencing SN activity. The volume of significant correlations between L-STR and SN was 1,936 mm^3^. R-STR–SN correlation volume was 4,480 mm^3^, substantially larger than in the left hemisphere. In contrast, the volume of significant R-MAT–SN correlations was 2,888 mm^3^. Comparing the total number of significant voxels, striosome-like seeds were 2.2-fold more likely to influence the SN than matrix-like seeds.

Regarding the overlap in rsFC within the SN, the DSC between left and right striosome-like seeds [(L-STR > L-MAT) ∪ (R-STR > R-MAT)] was zero. However, L-STR and R-MAT showed substantial overlap (DSC = 0.51). The overlap between L-STR and R-MAT was restricted to the MCC, suggesting that this area may be a unique convergence point that links the distinct connectivity patterns of striosome-like and matrix-like seeds with the SN.

### Frontoparietal network

The ipsilateral dominance for striosome-like seeds and contralateral dominance for matrix-like seeds was also evident in the FPN. For both L-STR and R-STR, connectivity with the FPN was largely ipsilateral (82.8 and 89.4% of significant connectivity, respectively). FPN regions with significant connectivity to striosome-like seeds (both left and right) included the MFG and IFG. In contrast, no significant differences were found for L-MAT. R-MAT–FPN connectivity was entirely contralateral (left MFG and IFG).

The volume of significant differences indicates that striosome-like seeds had a stronger influence on FPN activity than matrix-like seeds. The volume of significant correlations between L-STR and the FPN was 512 mm^3^, whereas no significant correlations were observed in L-MAT–FPN connectivity. In contrast, the R-STR–FPN correlations had a volume of 2,272 mm^3^, while the R-MAT–FPN correlations had a volume of 792 mm^3^. In combined hemispheres, striosome-like seeds were 3.5-fold more likely to influence the FPN than matrix-like seeds. The DSC for FPN regions was lower than for the DMN or SN: zero for L-STRÈR-STR, and minimal for L-STRÈR-MAT (DSC = 0.16). This overlap was restricted to the left IFG. While this overlap suggests that the left IFG may link connectivity between striosome-like and matrix-like networks, the substantially smaller overlap makes this association less clear for FPN than for regions in the DMN and SN.

### N-1 analysis: validating findings in the anterior insula

One cannot define striatal compartment identity based on connectivity to a region and subsequently quantify structural connectivity between that region and the striatal compartments—doing so distorts compartment-specific bias. We performed a *post-hoc* analysis to ensure that this potential source of distortion did not affect our rsFC results. Among our 10 compartment-favoring bait regions, the anterior insula was unique in also being an ROI for subsequent rsFC assessments. To ensure that our previously noted rsFC findings in the anterior insula were accurate (not distorted by its use as a bait region), we carried out a second striatal parcellation that left out the anterior insula: an N-1 parcellation. We then used these N-1 striosome-like and matrix-like seeds as the seeds for rsFC with the insula and compared these network maps to our original connectivity maps.

**FIGURE 9:**
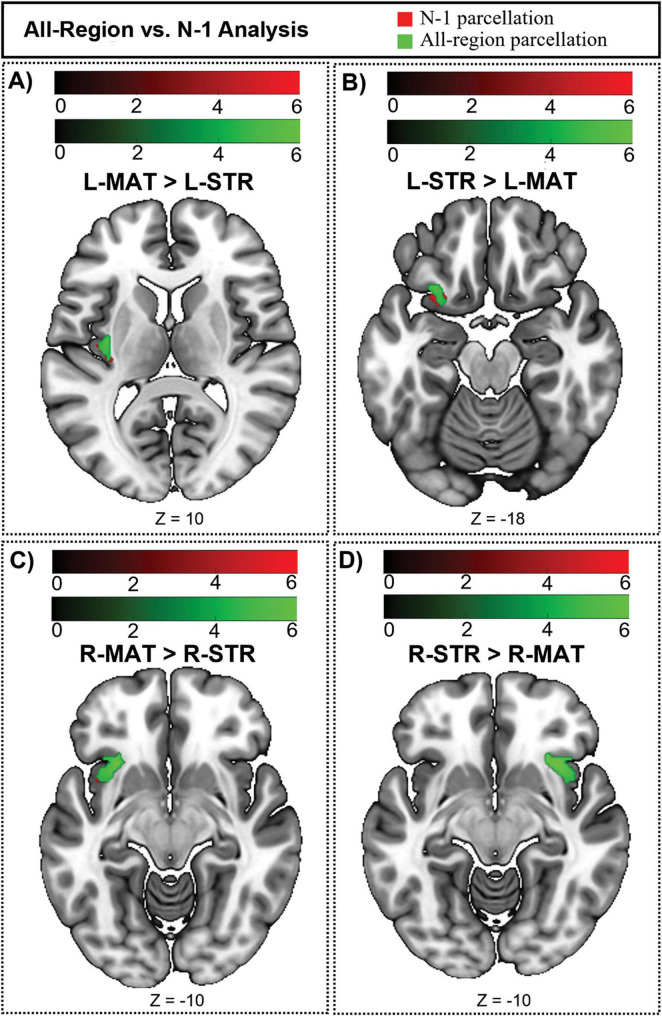
Compartment-specific bias in functional connectivity could be influenced by the use of the anterior insula as a bait region and subsequently as a site for whole-brain and salience network connectivity. Green voxels (original, all-region parcellation) are displayed in front of red voxels (N-1 parcellation). Connectivity is changed very little by leaving out the anterior insula. **(A)** The regions where the L-MAT exhibits stronger functional connectivity than the L-STR compartment. **(B)** Illustrates the regions where the L-STR has stronger connectivity than the L-MAT. **(C)** The regions where the R-MAT has stronger connectivity than the R-STR. **(D)** The regions where the R-STR has stronger connectivity than the R-MAT. The color bars correspond to *t*-values, with lighter colors reflecting higher *t*-values and stronger connectivity. Green = original parcellation (with anterior insula); Red = N-1 parcellation (leaving out the anterior insula). Images are displayed in anatomical convention, where the left hemisphere is shown on the left side of the image.

Our N-1 analysis was not meaningfully different from our original parcellation (Figure 9). The differences in cluster volume (N-1 *vs.* original) were minimal, ranging from a 0.26% decrease to a 0.43% increase. The overlap of N-1 and original clusters was high. The DSC (N-1 *vs.* original) for LMAT had moderate overlap of 0.61, while the R-MAT exhibited a higher DSC (0.85). Overlap within networks seeded by striosome-like voxels was high, with L-STR DSC of 0.75, and R-STR DSC of 0.89. Since the volume and location of compartment-specific significant clusters changed very little when parcellated without the anterior insula, we conclude that these compartment-specific patterns in rsFC were not distorted by the use of the anterior insula as a bait region for striatal parcellation.

## Discussion

For decades, the embryologic, pharmacologic, hodologic, and spatial segregation of striosome and matrix have suggested that the compartments’ functions are also distinct (Graybiel and Ragsdale, 1978; Brimblecombe and Cragg, 2017; Prager et al., 2020; Reiner et al., 2024).

Intriguing behavioral assessments in non-human primates (Canales and Graybiel, 2000; Karunakaran et al., 2021) and rodents (Smith and Graybiel, 2014; Friedman et al., 2015, 2017) are consistent with this hypothesis: striosome-specific activation is essential for mood- and stress-influenced learning and decision making. Reward appears to be a striosome-specific function as well, since electrical self-stimulation is reinforcing when electrodes are placed in striosome, but not in matrix (White and Hiroi, 1998). These striosome-specific functions are a small fraction of the tasks in which the striatum is involved, and do not attempt to map the implications of compartment-selective injury in human neuropsychiatric diseases (Crittenden and Graybiel, 2011). In this study, we began to explore the differences in rsFC between voxels with striosome-like and matrix-like patterns of structural connectivity, and mapped rsFC with both whole-brain and function-specific (triple) networks. Though we have previously demonstrated that striosome-like and matrix-like voxels are embedded in distinct cortico-striato-thalamo-cortical structural networks (Funk et al., 2023; Funk et al., 2024), to the best of our knowledge this is the first demonstration that the compartments occupy distinct functional networks.

Our findings suggest that compartment-specific functions in humans may be mediated by the influence of striosome or matrix acting within segregated functional networks. Moreover, these compartment-network relationships suggest that disease-specific symptoms may arise from selective decoupling of striosome or matrix from their segregated functional networks. While these insights into the rsFC of the striatal compartments are promising, our study has several limitations that must be considered.

First, the current method depends on the identification of striosome-like and matrix-like voxels using differential structural connectivity (probabilistic tractography). An important limitation of this approach is the incomplete mapping of compartment specific structural connectivity—many brain areas have never been assessed through injected tract tracers, including areas of great relevance to disorders of mood, motivation, and reward, such as the nucleus accumbens. The accuracy of our rsFC findings is contingent on how well these compartment-like seed masks represent striosome and matrix at the tissue level, despite the absence of histologic “ground truth” for regions that contribute to striatal function. We have previously demonstrated that striatal parcellation has a test-retest error rate of just 0.14% (Waugh et al., 2022), and tractography-based parcellation replicates the patterns of compartment-biased structural connectivity demonstrated through injected tract tracers in animals for cortical and subcortical regions (Figures 3–5; Waugh et al., 2022; Funk et al., 2023; Funk et al., 2024). However, our inferential method is not the equivalent of histologic identification of striosome and matrix. The complex three-dimensional structure of the striosome compounds this difficulty, since in any given plane of section one may sample the striosome along its axis, in cross section, or obliquely (Desban et al., 1989; Johnston et al., 1990; Brimblecombe and Cragg, 2017). Likewise, diffusion voxels may capture striosome branches that are waxing or waning within a given plane; quantifying the percentage of a voxel occupied by striosome or matrix is substantially more complex than measuring the cross-sectional area in histologic sections (Desban et al., 1993; Holt et al., 1997; Mikula et al., 2009). Our method emphasized high-confidence classifications based on strong bias toward one compartment. While this approach improved specificity, it reduced the contribution of transitional regions, potentially failing to identify striatal regions that did not conform to a binary compartment architecture. Future experiments could improve upon our method by using a probabilistic compartment assignment, rather than binary assignment as striosome- or matrix-like voxels. This may better reflect the overlapping and complex three-dimensional distribution of the compartments seen in histology. Future experiments to compare immunohistochemistry-based and MRI-based striatal parcellations in the same post-mortem brains are essential to validate and extend these methods, as has been demonstrated previously in the hippocampus (Iglesias et al., 2015) and thalamus (Iglesias et al., 2018). Until such experiments are completed, voxels identified through connectivity-based parcellation will remain only striosome- and matrix-*like*.

Second, the resolution of diffusion MRI is another important limitation of this method. The 1.25 mm isotropic voxels used here match the maximum diameter of the human striosome (Graybiel and Ragsdale, 1978; Holt et al., 1997), but smaller and obliquely sampled striosome branches are certain to produce partial volume effects and loss of discrimination between the compartments. At this and larger resolutions, every striosome-like voxel will include some fraction of matrix tissue. Inaccurate parcellation due to limited resolution could lead to imprecise seed voxels and yield incorrect functional connectivity maps. Voxel-based sampling may also obscure regional and tissue-type differences. While connectivity-based parcellation assumes a binary distinction between striosome and matrix, primate striata have regional variation in the histochemical markers for each compartment (Holt et al., 1997), cortico-striate projections are organized somatotopically (Goldman-Rakic, 1982, Donoghue and Herkenham, 1986, Berendse et al., 1988, Ragsdale and Graybiel, 1990, Eblen and Graybiel, 1995), and MSNs have greater transcriptomic diversity than is represented by the striosome-matrix binary (He et al., 2021). These limitations will be especially relevant in the study of human diseases with direct striatal pathology, such as Huntington disease (Tippett et al., 2007, Hedreen et al., 2024) or X-linked Dystonia Parkinsonism (Blood et al., 2017), where inter-compartment volume ratios and spatial relationships may be disordered. Although these limitations of resolution must be considered, the present study has a more precise resolution than our previous characterizations of compartment-like connectivity—where we demonstrated, in each of six distinct human MRI datasets, that striosome-like and matrix-like voxels follow the expected relative abundance, spatial segregation, intrastriate distribution, and biased structural connectivity demonstrated in animal and human histology (Waugh et al., 2022; Funk et al., 2023; Funk et al., 2024; Marecek et al., 2024; Waugh et al., 2025).

Third, this study has the potential limitation of relying on predefined brain networks rather than defining functional networks *de novo* directly from the current dataset. While predefined brain networks, such as those identified in standard atlases or by previous studies, offer a convenient reference framework, they may not capture the unique network configurations present in the current dataset. For example, the networks we assessed did not include subcortical nodes, which are among the most-important functional partners with the striatum. Identifying individualized networks may be especially important for studying the striatal compartments in populations with neuropsychiatric diseases or neurodevelopmental disorders. Likewise, rs-fMRI does not permit directional inference or task-specific functional mapping. Future research employing task-based paradigms or effective connectivity models may provide deeper insights into the distinct connectivity profiles and circuit-level functions of striosome- and matrix-like compartments, particularly with respect to subcortical structures with direct structural connectivity to the dorsal striatum, such as the substantia nigra, globus pallidus, and thalamus.

We observed that striosome-like seeds had markedly larger clusters of significant rsFC with the whole-brain and within the triple-network, compared to matrix-like seeds. Notably, striosome-like rsFC dominated even though the two compartment-like masks had equal volume and their distribution within the striatum was largely adjacent to the opposing compartment. These findings were highly dependent on the precise locations of these compartment-like voxels, as shifting their location by a few millimeters was sufficient to eliminate all differences in rsFC. This heightened functional connectivity may be attributable to the intrinsic properties of striosome MSNs, which have higher input resistance and greater depolarization than matrix MSNs (Miura et al., 2007), as well as lower thresholds for firing action potentials and higher firing frequencies (Crittenden et al., 2017). These properties suggest that striosome MSNs could be more readily excitable than matrix MSNs, facilitating their stronger influence on functional brain networks. Stronger rsFC in striosome-like voxels may also result from higher structural connectivity in these same voxels (48.6% larger number of streamlines in striosome-like bundles *vs.* matrix-like bundles, within-subjects comparison, Results 3.1). Recent demonstration that one can collect simultaneous rsFC and local field potentials in the rat striatum (Qiao et al., 2024) suggests that this intriguing approach could be used to investigate these compartment-specific differences in connectivity and functional activation. Such animal-based studies are needed to confirm these findings and to identify mechanisms underlying these differences.

Finally, while the HCP went to considerable lengths to exclude individuals with neurologic or psychiatric diagnoses—to the point of screening their parents and siblings for these diagnoses—the study was time limited. Subjects may have gone on to develop neuropsychiatric disorders at a later date, and subclinical or premanifest neuropsychiatric symptoms might influence the resting-state connectivity patterns we assessed, particularly within the triple-network. This limitation should be considered when interpreting our findings.

Previous studies investigating the striatal compartments have largely focused on animal models to explore aspects such as spatial distribution (Kubota and Kawaguchi, 1993), neurochemical composition (Holt et al., 1997), structural connectivity (Ragsdale and Graybiel, 1991), cortical organization (Ragsdale and Graybiel, 1990), functional segregation (Rajakumar et al., 1993), and differences in dopamine regulation (Perreault et al., 2011; Prager and Plotkin, 2019; McGregor et al., 2019). While these animal studies provided essential insights into compartment-specific differences, it is unknown whether human and animal functional networks support comparable behaviors. For example, while human and macaque have similar cortico-striate rsFC networks, the species differ substantially in the cortical regions that covary with the dorsal caudate (Liu et al., 2021). Understanding the specific roles of striosome and matrix in human brain function and their involvement in diseases requires investigations using human subjects. Human histological studies, both in non-diseased and diseased brains, have begun to yield insights into the unique roles the compartments may play in neuropsychiatric conditions (e.g., studies examining human brain tissue for striatal compartment pathology in disorders like Huntington disease or Parkinson disease; Vonsattel et al., 1985; Reiner et al., 1988; Kish et al., 1988; Gibb and Lees, 1991). However, studies of post-mortem tissue cannot investigate the dynamic functional properties of human brain networks.

The striatal compartments are functionally separable and play distinct roles in the brain’s processing of information and regulation of behaviors (Saka and Graybiel, 2003). One hypothesis proposes a functional differentiation between striosome and matrix MSNs, with the former being more involved in motivational aspects of behavioral function and the latter being more involved in sensory-motor functioning (Graybiel, 1990; Eblen and Graybiel, 1995; Graybiel, 1997). The striosome is involved in integrating limbic (emotional and motivational) information, modulating dopaminergic activity, and influencing reward-related behaviors. In contrast, the matrix primarily performs sensorimotor integration and motor control, receiving the majority of inputs from sensorimotor areas and regulating movement (Crittenden and Graybiel, 2011; Brimblecombe and Cragg, 2017; McGregor et al., 2019). These functional distinctions have largely been inferred from patterns of structural connectivity in animal studies. Intriguingly, selectively stimulating striosomal direct pathway neurons in mice leads to decreased speed of movement and reduced ambulatory turns toward the side of stimulation (Okunomiya et al., 2025), likely by inducing a widespread reduction in nigral dopamine release in the striatum. Indeed, D1/D2 projections from striosome MSNs are organized in the inverse manner of the classic direct and indirect pathways (Lazaridis et al., 2024): striosomal D1 projections directly inhibit dopaminergic neurons in the substantia nigra pars compacta, while striosomal D2 projections exert an indirect disinhibitory effect via a central zone of the globus pallidus externus. This dual pathway architecture may underpin the compartment-specific functional connectivity patterns we observed. These recent studies are a reminder that compartment-specific functions are not limited to remote, network-mediated effects; direct projections from each compartment rapidly and specifically modulate diverse behaviors in experimental animals. The findings of the current study provide evidence for widespread, compartment-specific differences in rsFC in healthy humans, affecting a range of functional networks involved in cognitive processing, decision-making, self-referential thinking, and coordinating responses to internal and external events—the DMN, SN, and FPN, respectively (Scolari et al., 2015; Wen et al., 2020; Schimmelpfennig et al., 2023). Combining our own findings with the insights provided by direct manipulation of the striosome in animals (Lazaridis et al., 2024; Okunomiya et al., 2025) underscores the importance of further investigating compartmental projection targets and their roles in goal-directed behavior, particularly in relation to neuropsychiatric conditions such as mood disorders, addiction, Huntington disease, and Parkinson disease, where compartment-specific dysfunction may explain particular clinical features of each disorder.

Hemispheric lateralization in the human brain is evident in key cognitive and motor tasks (Hervé et al., 2013) and is reflected in the ventral striatum as well, where the left and right hemispheres have distinct functional connectivity patterns. Zhang et al. (2017) found that connectivity with the left striatum was primarily to regions involved in self-control and internal processes, while the right striatum connected more with areas related to attention and external actions. In the present study, we found that the right striatum had larger compartment-specific rsFC biases than the left, for each of the networks we assessed. Likewise, we observed a robust distinction between the ipsilateral dominance of striosome-like connectivity and the contralateral dominance of matrix-like connectivity. This pattern was consistent across whole-brain and task-specific networks (DMN, SN, and FPN). These findings suggest a compartment-specific role in hemispheric processing, where striosomes may support the refinement of motor and cognitive processes on the same side of the body, possibly contributing to more localized, fine-tuned control (Fujiyama et al., 2019). In contrast, our results suggest that the matrix compartment facilitates inter-hemispheric communication, coordinating broader, bilateral movements and processes that require integration across hemispheres.

Our functional connectivity analyses revealed significant region-specific commonalities and differences, particularly in core triple-network nodes. Key regions such as the OFC, AIns, MCC, anterior ACC, and PCUN exhibited consistent connectivity differences between left and right, and between compartment-like networks (L-STR, L-MAT, R-STR, R-MAT), underscoring their critical roles in modulating cognitive control, emotional regulation, and self-referential processing within the triple-network (Botvinick et al., 2004; Rolls, 2004; Cavanna and Trimble, 2006; Buckner et al., 2008; Craig, 2009; Shackman et al., 2011; Uddin, 2015).

These areas are crucial nodes in the interaction between the DMN, SN, and FPN, suggesting that striosome and matrix compartments may differentially influence the balance between these networks, depending on the hemisphere and compartment involved. For instance, L-STR showed extensive connectivity in the left hemisphere, particularly in regions like the MTG, PCUN, AIns, MCC, and ACC. These areas are heavily involved in integrating sensory input with higher-order cognitive processes, such as decision-making and emotional responses, which are key functions of the SN and DMN. In contrast, L-MAT was associated with more limited regions, such as the sup-OFC, PIns, and HES in the left hemisphere, and the Inf-OFC in the right hemisphere. L-STR was connected with a broad array of regions while L-MAT had more restricted connectivity. R-STR demonstrated broad connectivity in the right hemisphere, including regions like the temporal gyrus (TG), MTP, STP, FG, and occipital regions (middle and inferior occipital gyri), along with the amygdala and cerebellum (lobule: VI, VII, VIII, IX, crus I, crus II), while also including left hemisphere regions like the SFG and MFG. The involvement of regions such as the TG, MTP, STP, FG, and occipital cortices, regions associated with auditory processing, visual processing, memory, and higher-order cognitive functions (Cabeza and Nyberg, 2000; Kanwisher et al., 2002; Grill-Spector and Malach, 2004; Hickok and Poeppel, 2007; Duncan, 2010). This suggests that R-STR may have a role in multimodal and associative sensory processing, while primary sensory processing is restricted to matrix.

The inclusion of left hemisphere regions in R-STR’s connectivity map indicates that it may play a role in cross-hemispheric regulation. This contralateral connectivity could be significant for tasks that require coordination between the hemispheres. Functional connectivity with the cerebellum suggests that R-STR might also influence motor, cognitive, or emotional tasks that require modeling and revision based on sensory and social feedback (Schmahmann, 2021; Zhang et al., 2023). On the other hand, R-MAT is notable for its extensive connectivity in the left hemisphere, encompassing regions such as TG, MTP, STP, IFG, AMYG, and FUS, with significant right hemisphere connectivity observed in the PIns and sup-OFC, supporting the lateralization of matrix-like networks, where RMAT preferentially engaged in interhemispheric rsFC and had distinct patterns of rsFC in each hemisphere. Although striosome and matrix MSNs have distinct projection targets reflecting their divergent roles in dopaminergic and motor-cognitive circuits, the cross-hemispheric mirroring we observed suggests they may participate in parallel but complementary processing streams. This organization may allow the striosome and matrix systems to coordinate different functional roles (e.g., emotion *vs.* cognition) across hemispheres, enabling integrated bilateral brain function despite their anatomical separation. This lateralization may underpin specialized functions in sensory, emotional, and cognitive processing. These findings highlight the nuanced and compartment-specific functional connectivity with the striatum, reflecting both shared and unique contributions to brain network dynamics.

Striosome-like connectivity dominated the triple networks (DMN, SN, and FPN), particularly in the right hemisphere. This was particularly true in the DMN, which is primarily involved in self-referential thinking, episodic memory, and cognitive-sensory integration (Dixon et al., 2022). The lateralization of DMN connectivity may suggest that in the right hemisphere, the striosome plays a critical role in integrating spatial and sensory information, potentially enhancing visuospatial processing and social cognition. In the SN, which filters and prioritizes salient stimuli (Menon and Uddin, 2010; Uddin, 2015), the more extensive right hemisphere striosome connectivity with the AIns and ACC underscores its potential contribution to emotional regulation (Coen et al., 2009; Stevens et al., 2011) and cognitive control (Sterzer and Kleinschmidt, 2010). This hemispheric bias could imply a specialization of the striosome for processing emotional and sensory information within the SN. Finally, in the FPN, which governs executive functions and decision-making, the ipsilateral dominance of striosome connectivity, particularly in frontal cortices, suggests that it may play a key role in lateralized cognitive processes such as sequential reasoning and motor planning in the left hemisphere (Corballis, 2014), while the right hemisphere supports visuospatial tasks (Hartikainen, 2021). These findings point to a lateralized, compartment-specific modulation of higher-order cognitive functions across the triple networks, with the right striosome exerting a broader influence on network dynamics than matrix.

Interestingly, though striosome-like and matrix-like rsFC was markedly divergent by hemisphere, our most- and least-biased networks (L-STR and R-MAT) partially overlapped (DSC = 0.23). This overlap suggests that despite the distinct functional connectivity patterns typically observed between striosome-like and matrix-like networks, there are limited areas where left and right hemisphere networks may impinge upon the other compartment. The regions of overlap (all left hemisphere) include the anterior insula, anterior and middle cingulate cortices, precuneus, and orbitofrontal cortex. These regions are key nodes in the salience and default mode networks that are critical for emotional processing, cognitive control, and self-referential thinking (Dixon et al., 2022; Schimmelpfennig et al., 2023). This convergence may reflect a level of integration between the striosome and matrix compartments, where they jointly contribute to key cognitive and emotional processes. The shared connectivity in these areas could be essential for coordinating complex behaviors that require the integration of emotional, cognitive, and self-referential information, underscoring the central role of compartment-specific functional networks.

## Conclusion

In conclusion, this study provides strong evidence that in humans, the striosome and matrix compartments operate within segregated functional networks. Each compartment’s functional networks are organized differently (ipsilateral *vs.* contralateral; global *vs.* local). Striosome-like voxels primarily show ipsilateral connectivity and appear to be involved in hemisphere-specific processing. In contrast, matrix-like voxels exhibit stronger contralateral connectivity, indicating a more global role that integrates information across hemispheres. These patterns of connectivity suggest that striosome may be more specialized for local processing within the same hemisphere, whereas matrix compartments may contribute to broader, cross-hemispheric network dynamics. This distinction between “global” and “local” network organization helps to further elucidate the differential roles the striatal compartments play in brain function. Striosome-like seeds exhibited widespread functional connectivity with key nodes of the default mode, salience, and frontoparietal networks, suggesting involvement of the striosome in higher-order cognitive functions, emotional regulation, and integrative processing. In contrast, matrix-like seeds demonstrated more limited connectivity within these networks, potentially reflecting its specialized role in sensorimotor integration and motor control (Deng et al., 2015; Graybiel and Matsushima, 2023). Task-based fMRI could be utilized to assess the activation of compartment-like seeds during sensory, motor, limbic, and executive tasks. While the temporal pattern of activation with tasks is an essential part of understanding the function of striosome and matrix, such investigations are beyond the scope of the current study.

Each striatal compartment is embedded in segregated intrinsic functional networks, providing an anatomic substrate for striosome and matrix to have distinct functional roles. The functional architecture of the striatum is intricate and highly localized—slight shifts in the location of striatal seeds eliminated all rsFC—suggesting that particular functions may also localize to limited zones within striosome or matrix. Future compartment-specific interventions may also require this level of anatomic precision. Linking specific symptoms with compartment-specific alterations in functional connectivity may suggest novel neuropathological mechanisms underlying neuropsychiatric disorders associated with striatal dysfunction, such as Parkinson disease (Albin et al., 1989), Huntington disease (Rosenblatt and Leroi, 2000), or obsessive-compulsive disorder (Burguière et al., 2015). Mapping the contributions of each striatal compartment to specific functional networks is essential for identifying the functions of striosome and matrix.

## Data Availability

Publicly available datasets were analyzed in this study. This data can be found here: ConnectomeDB, the portal for accessing data from the Human Connectome Project (https://db.humanconnectome.org).

## Footnotes

1
https://www.fil.ion.ucl.ac.uk/spm/software/spm12/
